# Vestibular Organ and Cochlear Implantation–A Synchrotron and Micro-CT Study

**DOI:** 10.3389/fneur.2021.663722

**Published:** 2021-04-07

**Authors:** Hao Li, Nadine Schart-Moren, Gunesh Rajan, Jeremy Shaw, Seyed Alireza Rohani, Francesca Atturo, Hanif M. Ladak, Helge Rask-Andersen, Sumit Agrawal

**Affiliations:** ^1^Department of Surgical Sciences, Otorhinolaryngology and Head and Neck Surgery, Uppsala University, Uppsala, Sweden; ^2^Section of Otolaryngology, Head and Neck Surgery, Uppsala University Hospital, Uppsala, Sweden; ^3^Department of Otolaryngology, Head & Neck Surgery, Luzerner Kantonsspital, Lucerne, Switzerland; ^4^Department of Otolaryngology, Head & Neck Surgery, Division of Surgery, Medical School, University of Western Australia, Perth, WA, Australia; ^5^Centre for Microscopy, Characterization and Analysis, Perth, WA, Australia; ^6^Department of Otolaryngology-Head and Neck Surgery, Western University, London, ON, Canada; ^7^Department of Otolaryngology, University of Sapienza, Rome, Italy; ^8^Department of Medical Biophysics and Department of Electrical and Computer Engineering, Western University, London, ON, Canada

**Keywords:** human, synchrotron, micro-CT, vestibular organ, cochlear implant

## Abstract

**Background:** Reports vary on the incidence of vestibular dysfunction and dizziness in patients following cochlear implantation (CI). Disequilibrium may be caused by surgery at the cochlear base, leading to functional disturbances of the vestibular receptors and endolymphatic duct system (EDS) which are located nearby. Here, we analyzed the three-dimensional (3D) anatomy of this region, aiming to optimize surgical approaches to limit damage to the vestibular organ.

**Material and Methods:** A total of 22 fresh-frozen human temporal bones underwent synchrotron radiation phase-contrast imaging (SR-PCI). One temporal bone underwent micro-computed tomography (micro-CT) after fixation and staining with Lugol's iodine solution (I_2_KI) to increase tissue contrast. We used volume-rendering software to create 3D reconstructions and tissue segmentation that allowed precise assessment of anatomical relationships and topography. Macerated human ears belonging to the Uppsala collection were also used. Drilling and insertion of CI electrodes was performed with metric analyses of different trajectories.

**Results and Conclusions:** SR-PCI and micro-CT imaging demonstrated the complex 3D anatomy of the basal region of the human cochlea, vestibular apparatus, and EDS. Drilling of a cochleostomy may disturb vestibular organ function by injuring the endolymphatic space and disrupting fluid barriers. The saccule is at particular risk due to its proximity to the surgical area and may explain immediate and long-term post-operative vertigo. Round window insertion may be less traumatic to the inner ear, however it may affect the vestibular receptors.

## Introduction

There are various reports on the incidence of vestibular dysfunction and vertigo following cochlear implantation (CI) in adults and children. Although CI is considered to be safe, the traumatic action of electrode insertion into the cochlea risks impairing vestibular function. Seriously incapacitating vertigo is rare, and there is usually complete resolution (1). Different factors have been ascribed as possible causes, such as labyrinthine status before CI surgery or concurrent inner ear disease. Older patients and patients with preoperative dizziness may be more prone to vestibular injury, and this may occasionally be associated with tinnitus and fluctuating hearing loss (2–5). Dizziness may be experienced directly after surgery or with delayed onset (6). In some instances, endolymphatic hydrops (EH) may be suspected (7). Therefore, vestibular impairment can be influenced by surgical impact, patient age, and cause of deafness.

The human ear contains five end-organs, each of which can be affected by surgery at the cochlear base or by electrode insertion itself. Postmortem histopathological studies of the temporal bones of CI recipients have reported significant structural changes in end-organs, including the saccule, the utricle, and the semicircular canals (8, 9). Injury of cochlear and vestibular tissue may lead to the mixing of fluids and the alteration of otolith membranes and receptor cells. CI may damage the lateral cochlear wall disturbing endolymph homeostasis leading to cochlear hydrops. CI may also obstruct endolymph flow between the cochlea and the saccule by blocking the reunion duct (RD) or cochlear duct causing cochlear hydrops and collapse of the saccule (9). Long-term changes may occur from inflammation, fibrosis, and ossification (8). There is a particular risk of damage to the saccule, which is located in the spherical recess close to the base of the cochlea and round window (RW). Moreover, the main cochlear vein is located in the floor of the scala tympani (ST) near the final position of the CI electrode.

Non-invasive, high-resolution synchrotron radiation and 3D imaging of temporal bone specimens have earlier been performed (10). To improve soft tissue contrast, chemical staining was also introduced to visualize the hearing organ and nerve elements using absorption based synchrotron imaging (11, 12). This necessitates opening of the windows of the inner ear with risk for artifact generation. In lieu of staining, synchrotron radiation phase-contrast imaging (SR-PCI) can be used to increase visualization of soft tissues. This technique exploits x-ray intensity variations to produce edge contrast thereby improving soft tissue visualization. At the same time, SR-PCI conserves visualization of bone while avoiding the artifacts introduced with staining, sectioning, and decalcification used in histopathology (13–15). Elfarnawany et al. first performed SR-PCI on intact human cochleae to obtain 3D reconstructions of cochlear soft tissues (16). The high-resolution scans obtained through this technique were capable of revealing cytoarchitecture similar to histology (17, 18). Subsequent groups have applied the SR-PCI technique to other parts of the temporal bone, including the middle ear and ossicles (19, 20). Recently, Anschuetz et al. demonstrated synchrotron radiation imaging of the human auditory ossicles at the sub-micron level (21).

The present study aimed to three-dimensionally analyze the intricate anatomy of the surgical region to optimize atraumatic approaches in CI to limit the surgical impact on the vestibular apparatus and associated neural pathways. A total of 22 fresh human temporal bones underwent SR-PCI and one fresh bone underwent micro-computed tomography (micro-CT) after fixation and staining with Lugol's iodine solution (I_2_KI) to increase tissue contrast. In addition, we analyzed the archival temporal bone collection in Uppsala described in earlier investigations (22, 23). Different cochleostomies (COs) were made with metric analyses. Volume-rendering software was then used to create three dimensional (3D) reconstructions allowing tissue segmentation and detailed assessment of anatomical relationships, metric analyses, and topography. It was found that the RW surgical approach may be preferred to limit the risk for vestibular dysfunction and vertigo after CI, assuming there are no anatomical restrictions preventing this approach.

## Materials and Methods

### Ethical Statements

#### Human Temporal Bones

Twenty-two adult human cadaveric cochleae were used in this study. Specimens were obtained with permission from the body bequeathal program at Western University, London, Ontario, Canada, in accordance with the Anatomy Act of Ontario and Western's Committee for Cadaveric Use in Research (approval no. 06092020). Ethics approval for the micro-CT project was obtained from the University of Western Australia (UWA, RA/4/1/5210**)**, and the human temporal bones were provided by the Department of Anatomy at UWA.

The adult cadaveric temporal bones were fresh-frozen and then fixed in 3.7% formaldehyde and 1% glutaraldehyde in phosphate buffer for 5 days. The bones were thawed and cut to a sample (40 mm diameter, 60 mm length) from each temporal bone. All samples were cut from the middle ear toward the inner ear. The tissue was rinsed and dehydrated in a graded ethanol series. No staining, sectioning, or decalcification was performed on the specimens.

#### SR-PCI and Imaging Technique

The SR-PCI technique used in the present investigation was recently described by Elfarnawany et al. (16) and Koch et al. (13). Each sample was scanned using SR-PCI combined with CT at the Bio-Medical Imaging and Therapy (BMIT) 05ID-2 beamline at the Canadian Light Source, Inc. (CLSI) in Saskatoon, SK, Canada. The imaging field of view was set to 4,000 × 950 pixels corresponding to 36.0 × 8.6 mm, and 3,000 projections over a 180° rotation were acquired per CT scan. CT reconstruction was performed, and the 3D image volume had an isotropic voxel size of 9 μm. The acquisition time to capture all projections per view was ~30 min. For 3D segmentations of the cochlear anatomy, structures were traced and color-labeled manually on each SR-PCI CT slice (approximately 1,400 slices per sample). The open source medical imaging software, 3D Slicer version 4.10 (24), was used to create detailed 3D representations of the basilar membrane (BM), spiral ganglion (SG), and connective dendrites between these structures, which allowed for accurate delineation when compared with traditional two-dimensional (2D) slices. Measurements were made in 22 temporal bones by two independent observers. Distances from the utricle macula, posterior semicircular canal ampulla, saccule macula, and saccule membrane to the middle of the RW were assessed.

#### Micro-CT

Micro-CT was used to analyze the 3D anatomy of the nerves in the internal acoustic meatus. We used a diffusible iodine-based technique to enhance contrast of soft tissues for diffusible iodine-based contrast-enhanced computed tomography (dice-CT) (25). Increased time penetration of Lugol's iodine (aqueous I_2_KI, 1% I_2_, 2% KI) offers possibilities to visualize between and within soft tissue structures (25). The temporal bone was fixed in a modified Karnovsky's fixative solution of 2.5% glutaraldehyde, 1% paraformaldehyde, 4% sucrose, and 1% dimethyl sulfoxide in 0.13 M of Sorensen's phosphate buffer. Soft tissue contrast was achieved by staining the sample for 14 days, as described by Culling et al. (26). X-ray micro-CT was conducted using a Versa 520 XRM (Zeiss, Pleasanton, CA, USA) running Scout and Scan software (v11.1.5707.17179). Scans were conducted at a voltage of 80 kV and 87 μA, using the LE4 filter under 0.4 × optical magnification and a camera binning of 2. Source and detector positions were adjusted to deliver an isotropic voxel size of 23 μm. A total of 2,501 projections were collected over 360°, each with an exposure time of 1 s. Raw projection data were reconstructed using XM Reconstructor software (v10.7.3679.13921; Zeiss) following a standard center shift and beam hardening (0.1) correction. The standard 0.7 kernel size recon filter setting was also used.

### Uppsala Temporal Bone Collection

We used the archival human temporal bones from autopsies and 324 plastic and silicone molds described in earlier publications (22, 23). The collection was established during the 1970s and 1980s at the Department of Diagnostic Radiology and Otolaryngology at Uppsala University Hospital (27, 28). All bones and molds underwent micro-CT as described earlier (23). The topographic anatomy of the “hook” region with relationships between the oval window (OW), RW, osseous spiral lamina (OSL), and spiral ligament (SL) were examined and photographed as described earlier by Atturo et al. (29). Different sized cochleae were analyzed and conventional anterior (ACOs), antero-inferior (AICOs), and inferior COs were made, including the enlarged RW approach (30, 31). The proximity of various COs to the vestibular organ was studied, both from “inside” and “outside” the labyrinth.

## Results

**Figure 1:**
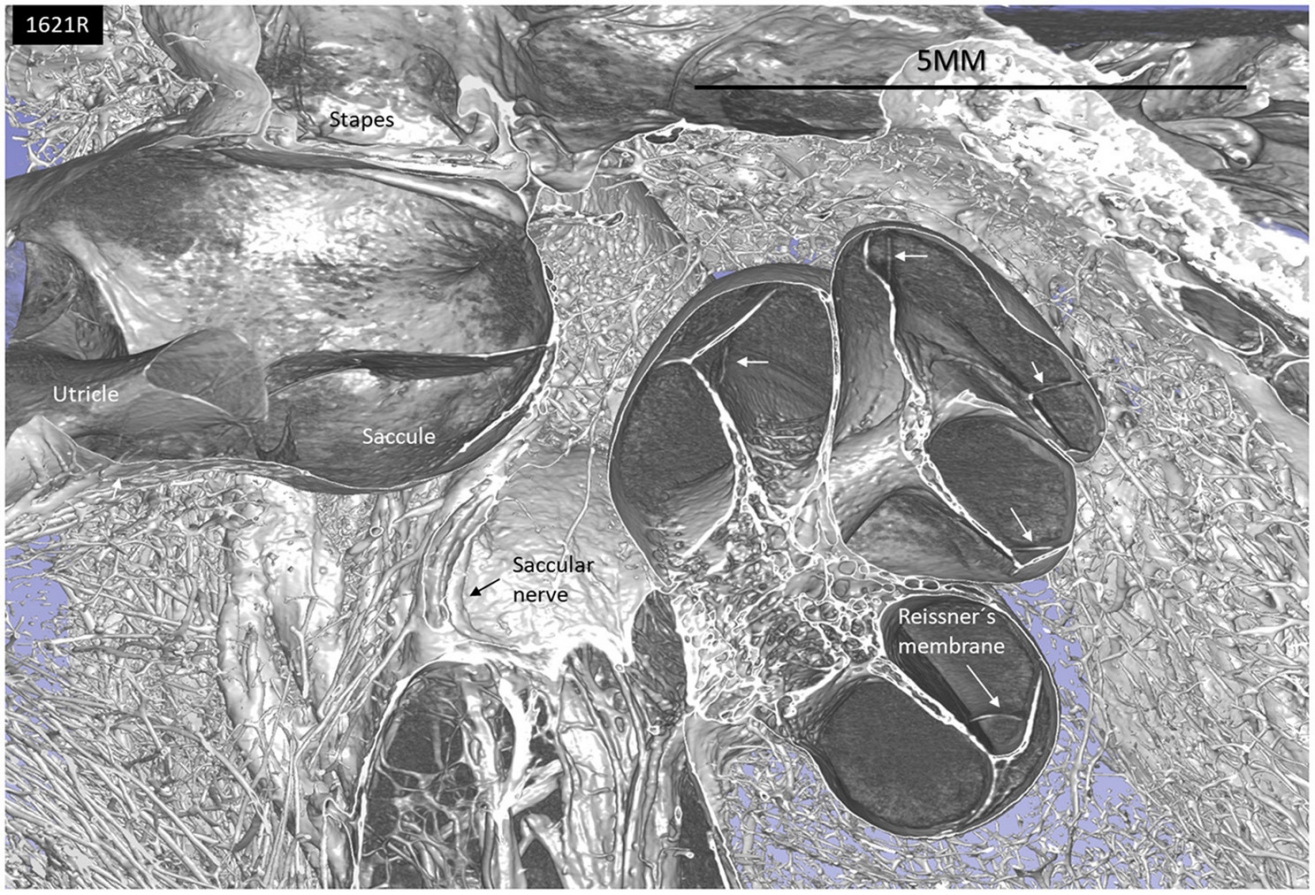
SR-PCI and 3D reconstruction of a left human inner ear (superior view) using 3D slicer (version 4.10; www.slicer.org). The cochlea, utricle, saccule, and saccular nerve are seen with cranial nerves in the fundus of the IAC.

SR-PCI and micro-CT with contrast enhancement reproduced both the soft and bony tissue of the human cadaver labyrinth. A notable 3D reproduction of the membranous labyrinth in a left human temporal bone is shown in Figure 1. The cochlear and vestibular nerves and their branches could be followed from the internal acoustic canal (IAC) to the peripheral organs.

**Figure 2:**
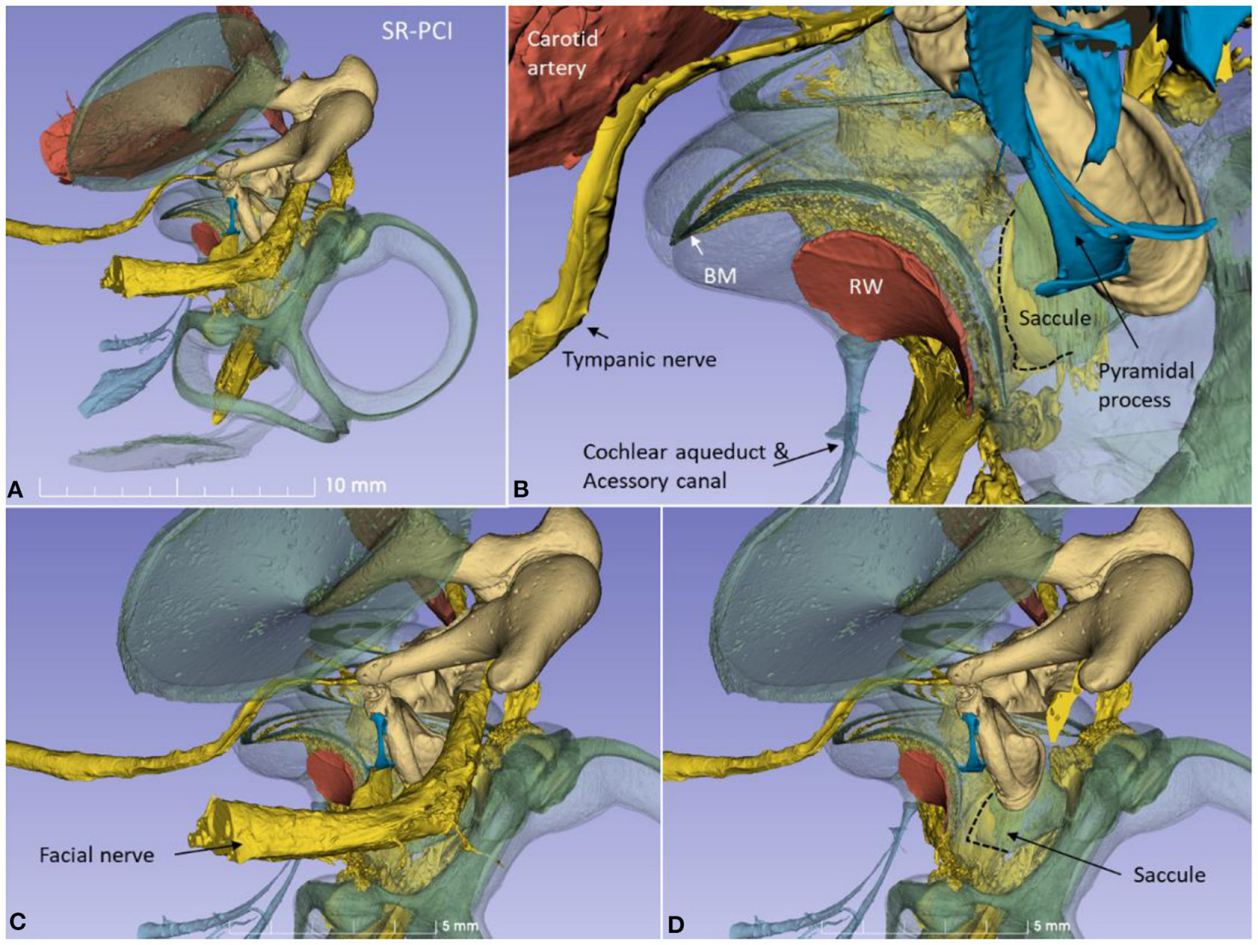
**(A)** SR-PCI 3D modeling of a left human temporal bone with a surgical view through the facial recess. **(B)** The relationship between the RW and the saccule is seen. The cochlear aqueduct (CA) and a second accessory canal are seen. **(C,D)** show the facial recess anatomy with **(C)** and without **(D)** the facial nerve.

The 3D modeling shows the surgical anatomy through the facial recess (Figure 2). The anatomical details of the cochlear base are visualized together with the saccule and utricle. Removal of the facial nerve demonstrates the close relationship between the cochlea and the saccule.

**Figure 3:**
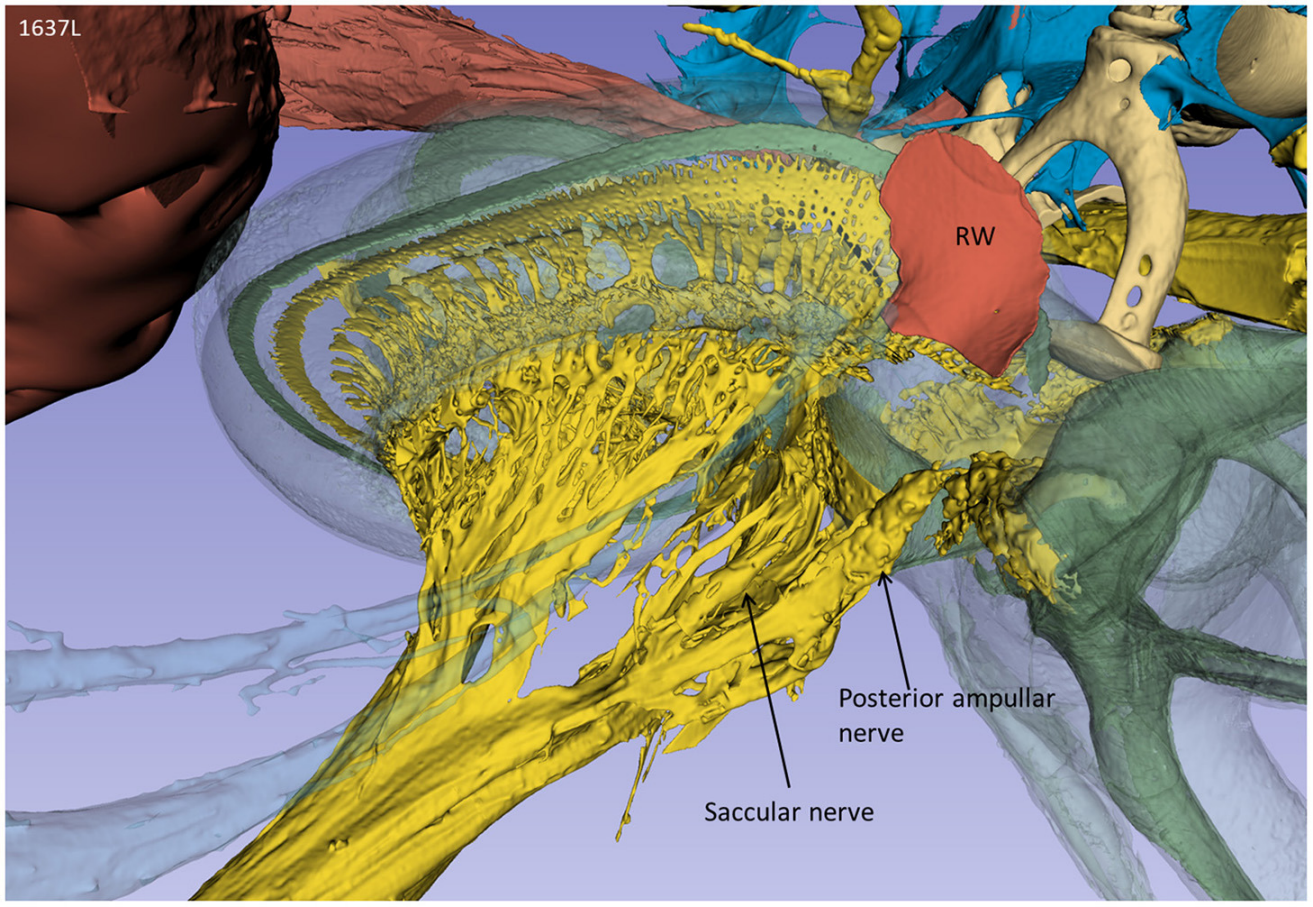
A posterior-inferior view of the specimen shown in Figure 2. The relationships between the RW and the posterior ampulla and saccular nerves are shown. The distance between the middle of the RW and the middle part of the posterior ampulla was 2.6 mm.

From an inferior angle, the relationship between the RW and the saccular and posterior ampulla nerves is shown (Figure 3).

**Figure 4:**
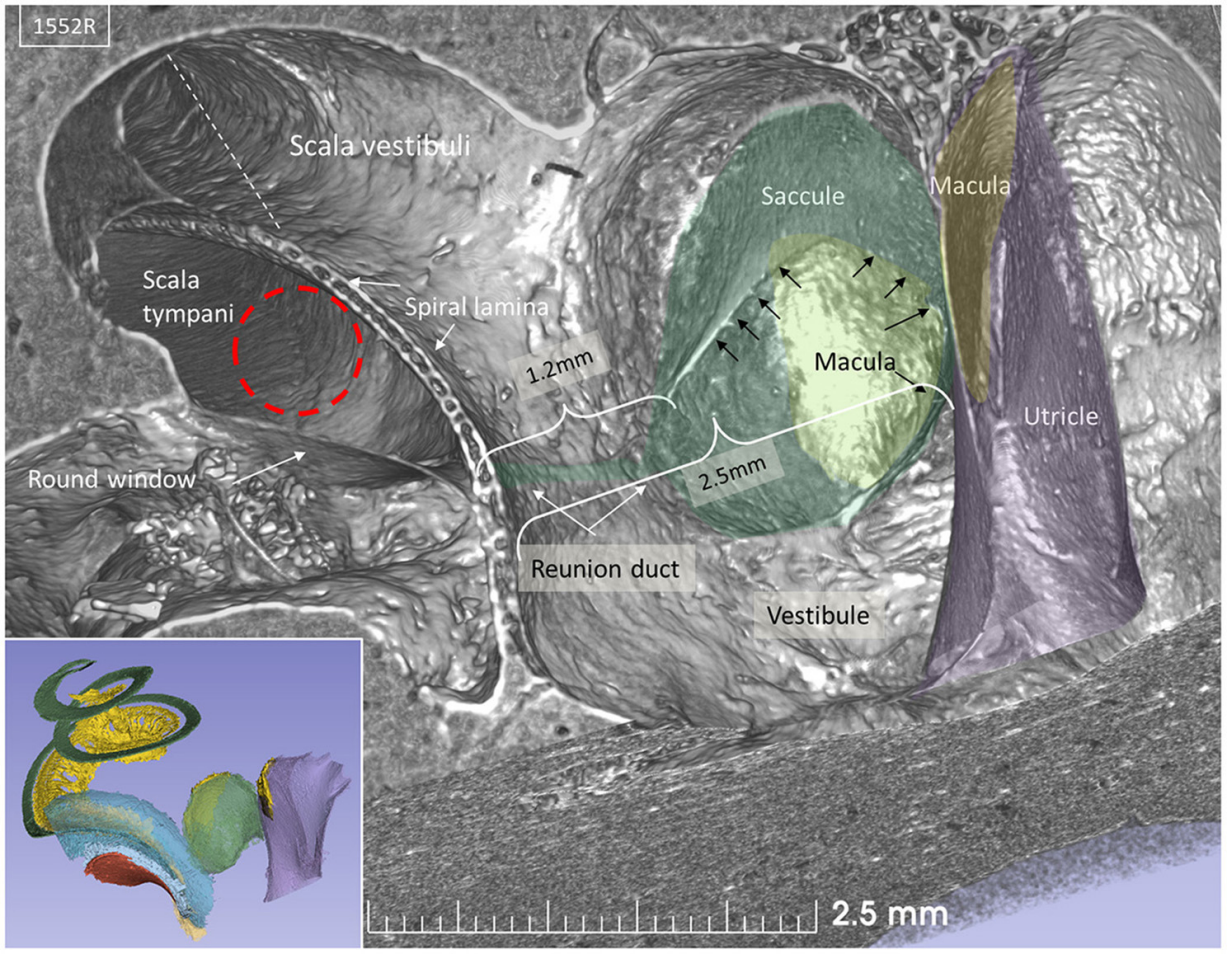
SR-PCI section at the level of the RW and vestibule (lateral view). The RW and the position of a virtual electrode (dashed red line) are shown. The saccule lies in the spherical recess in the medial bony wall of the vestibule. It consists of a thicker and thinner part limited by thicker bands (arrows). The macula is stained yellow. The position of the RD is shown. Inset shows the modeled 3D anatomy with the saccule, RW (red), and spiral ligament of the cochlear base (blue). The broken line represents Reissner's membrane.

Lateral sectioning at the cochlear base of a left ear demonstrates the relationship between the saccule and utricle and the ST in more detail (Figure 4). Electrode insertion near the posterior corner of the RW and at an acute angle may jeopardize the OSL with consequences of entering the vestibule. The RD lies on the superior edge of the SL and connects the scala media and saccule. The RD is challenged if the bony lamina is perforated. The mean distance between the mid-portion of the RW and the saccule was 2.66 mm (*SD* = 0.35 mm) and between the RW and the saccule macula was 3.21 mm (*SD* = 0.29 mm). The mean distance between the RW and the utricle macula was 3.79 mm (*SD* = 0.32 mm) (Supplementary Table 1).

The saccular wall consists of both a thick and a thin part. The two parts are separated by a thickening in the membrane. The thin part faces the middle ear, while the thick part reinforces the saccule against the spherical recess. The thin part was difficult to reproduce three-dimensionally and gave the impression of an imperfection in the wall.

**Figure 5:**
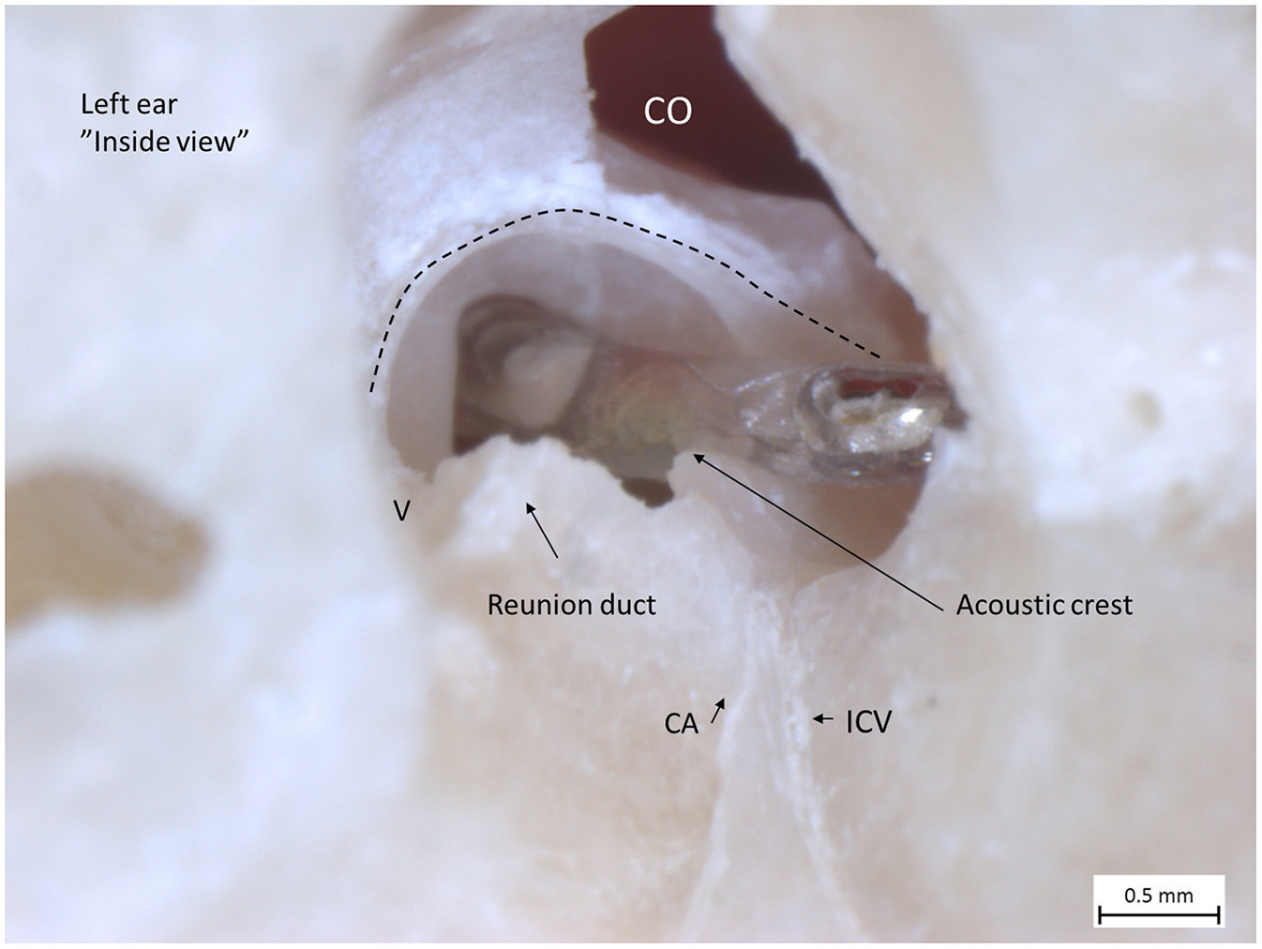
Left human micro-dissected temporal bone (taken from the temporal bone collection of Uppsala Museum) shows the RW and acoustic crest from “inside” the labyrinth. The OSL was resected, with the secondary lamina partially preserved along the rim of the RW. The broken line shows the attachment of the BM around the RW. The electrode was inserted via the RW. It rides upon the acoustic crest before reaching the ST. An anterior CO was drilled. The CA and the inferior cochlear vein (ICV) channels were dissected as well as the RD.

**Figure 6:**
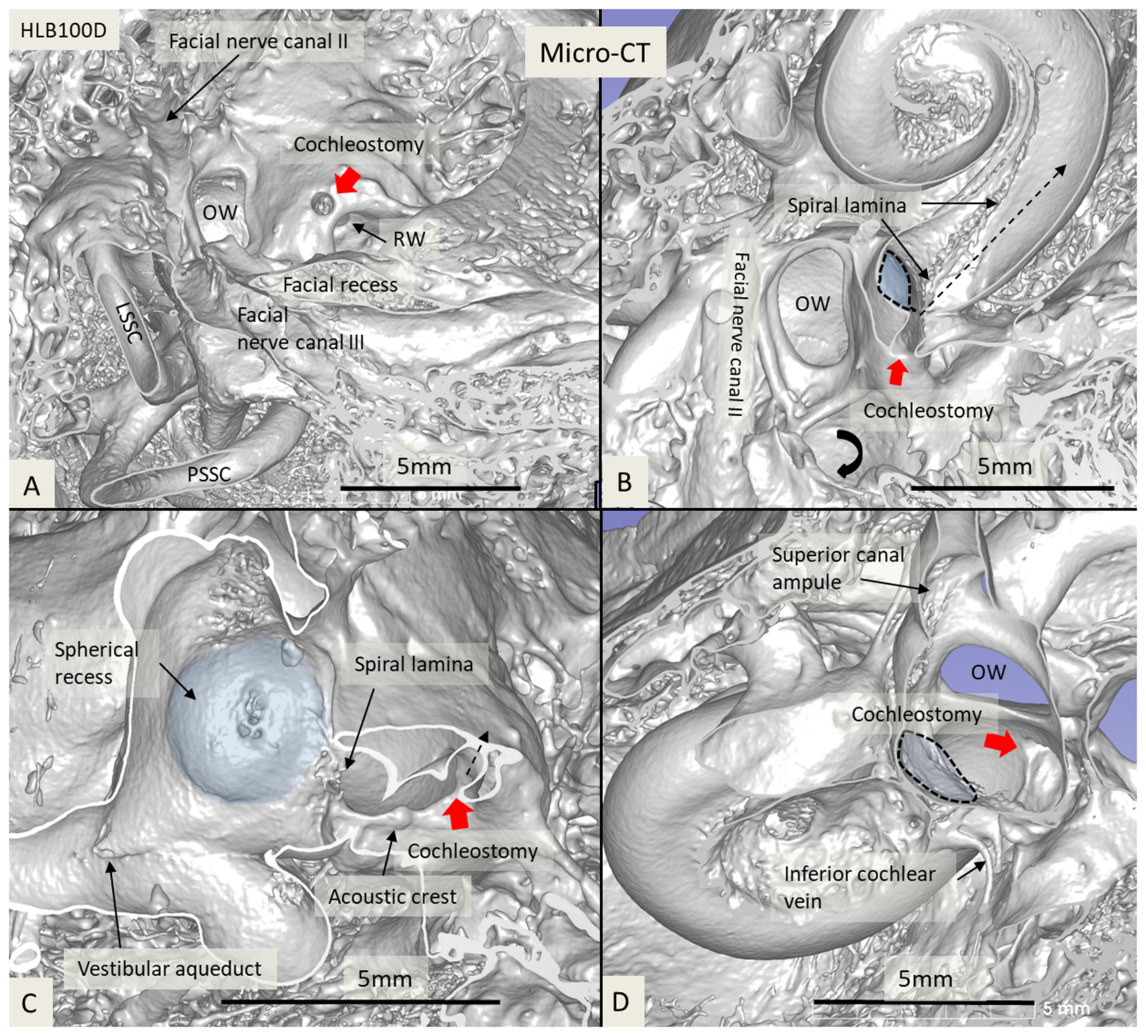
Micro-CT and 3D reconstruction of a macerated human temporal bone (right ear). An anterior CO was made (red arrow), and its relation to the spherical recess (blue) and saccule was studied. **(A)** Surgical view shows the CO and the RW. **(B)** Lateral cropping demonstrates the entrance canal of the CO and its relation to the spherical recess. The bold arrow shows the tympanic sinus. **(C)** Medial cropping shows the spherical recess (blue) with bony foramina of the saccular nerve. The broken arrow shows the direction of the scala tympani. **(D)** Medial view shows CO and spherical recess (dashed line). OW, oval window; RW, round window; LSSC, lateral semicircular canal; PSSC, posterior semicircular canal.

**Figure 7:**
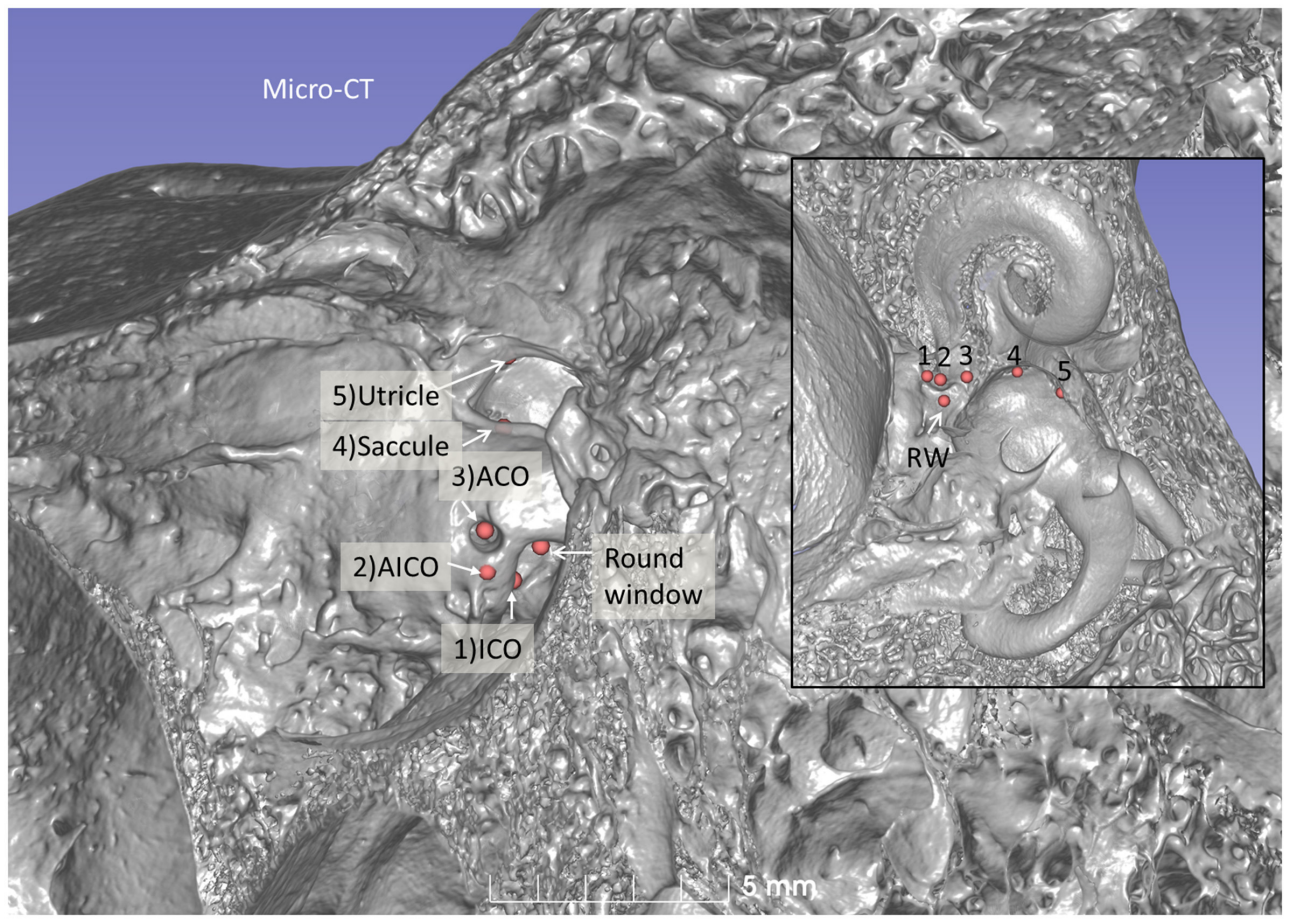
Micro-CT 3D reconstruction of a left human temporal bone (lateral view). An ACO (3) was made, and the distances to the saccular (4) and utricular (5) nerve foramina can be assessed (inset). Virtual AICO (2) and ICO (1) are shown with fiducials.

The macerated human ears revealed extensive anatomic variations of the basal or “hook” region of the cochlea. Drilling and insertion of a CI electrode via an anterior or anterior-inferior CO invariably damaged cochlear structures. Membrane rupture may lead to a mixture of fluids, and bone dust potentially contaminates the vestibule with risk for damage to the vestibular receptors. The soft tissue suspending the BM along the rim of the RW varied among individuals, and even an inferiorly located CO occasionally damaged cochlear tissues. A larger distance between the OW and RW seemed to diminish the risk for mechanical trauma to the SL at inferior CO drilling. Smaller cochleae increased the risk of injuring the SL by leading to a direct trajectory to the saccule. A RW inserted electrode is visualized in Figure 5, from “inside” the labyrinth. Distances from the utricle macula, saccule macula, and saccule membrane to the middle of the RW were measured in all 22 temporal bones and are shown in a box plot. The distances from different COs to the utricular and saccular macular nerve foramina were also assessed (Figures 6, 7).

**Figure 8:**
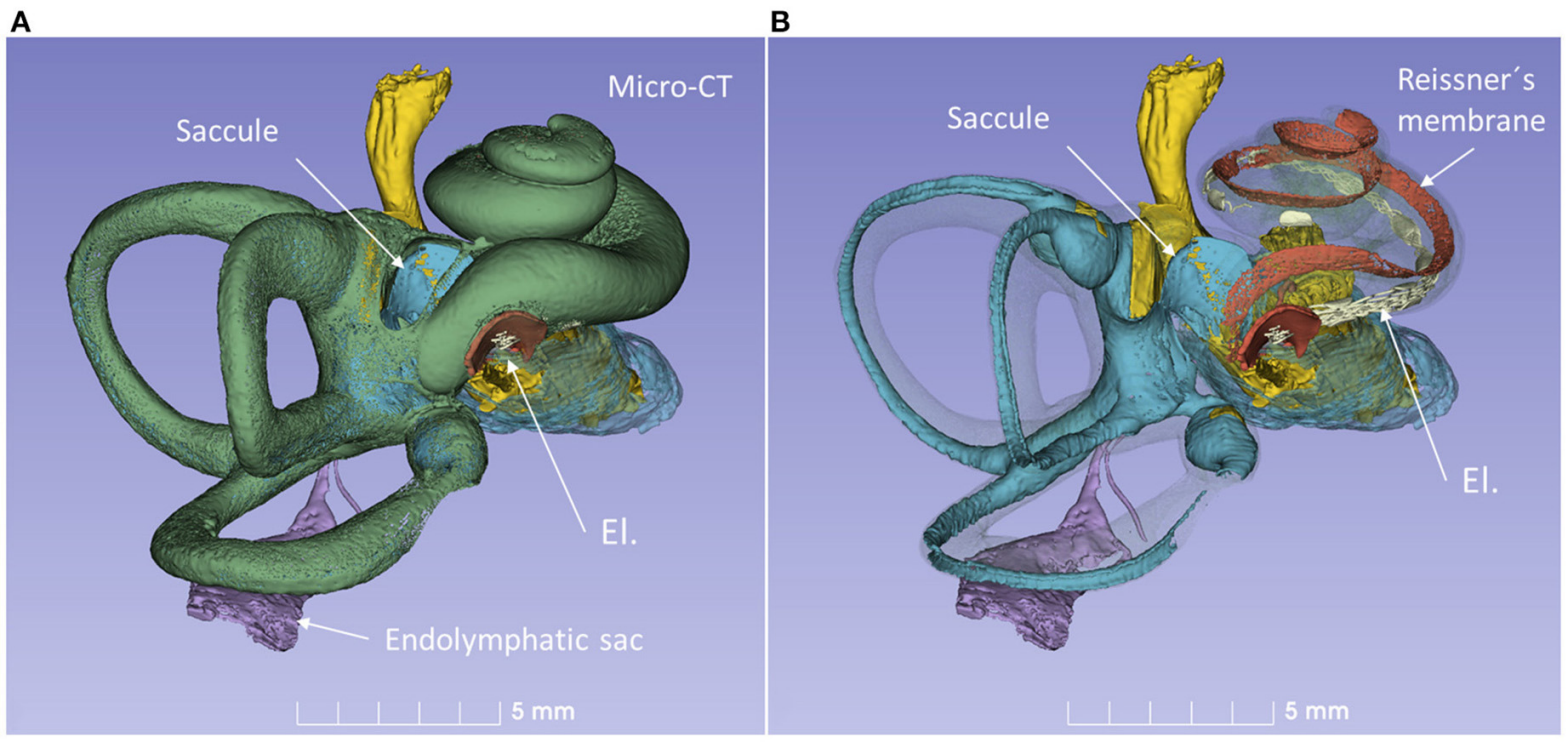
3D model of a right human temporal bone from micro-CT. Increased penetration time of aqueous I_2_KI improved visualization of soft tissue structures. The cochlea was virtually implanted with an electrode (El) through the RW. **(A)** With bone capsule. **(B)** Bone capsule was made transparent to visualize the inner ear soft tissues.

**Figure 9:**
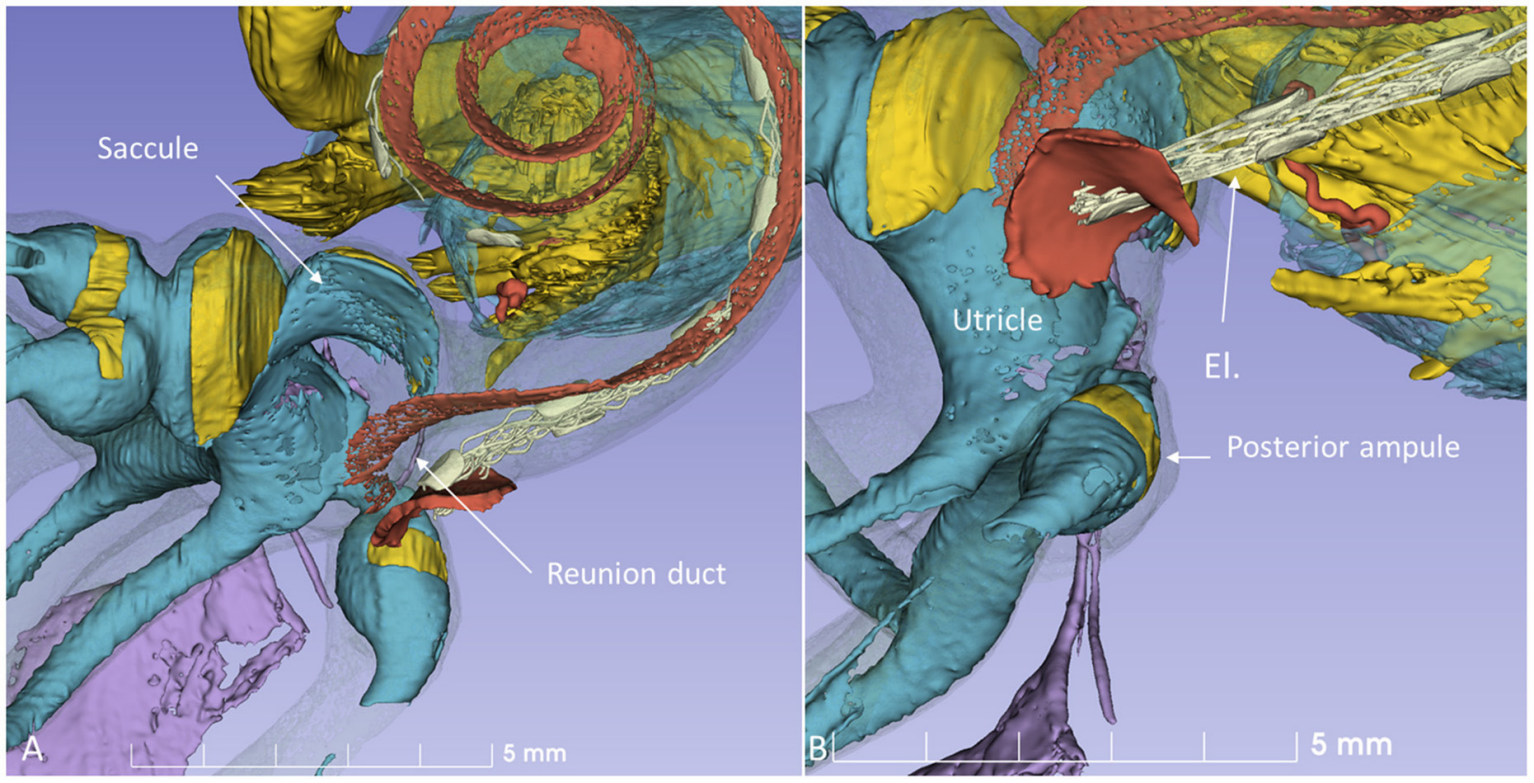
3D model in Figure 8 is shown at higher magnification. **(A)** The modeled RD is seen. **(B)** The posterior ampule and its relation to the RW can be seen. El, cochlear implant electrode.

A virtual CI surgery using the RW approach in a 3D reconstructed human temporal bone from a micro-CT is demonstrated in Figures 8, 9. The position of the saccule is seen after the bony capsule was made transparent (Figures 8A,B). The lateral wall of the saccule is visualized through the OW, reaching cranially to the floor of the utricle. The inferior cochlear and saccular veins in the floor of the ST were found to be at low risk for damage.

## Discussion

**Figure 10:**
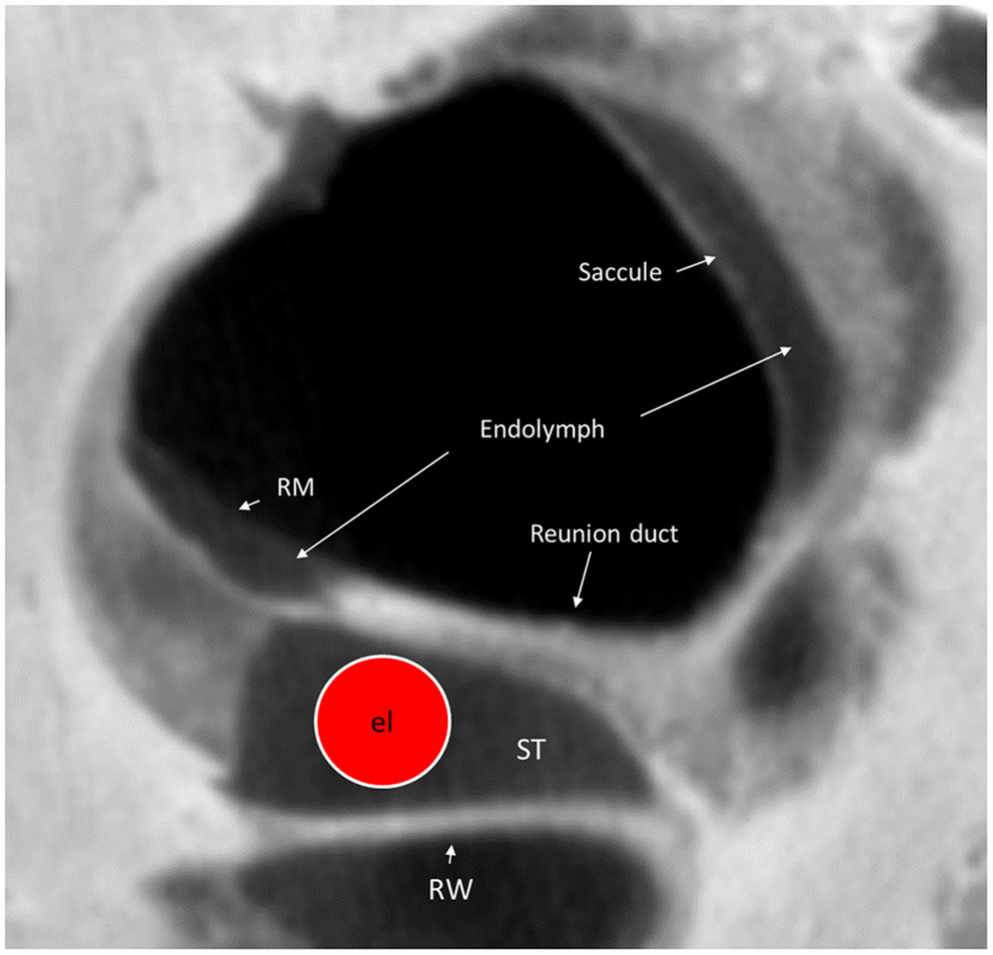
Micro-CT cross-section of the cochlear base at the RW. A virtual CI electrode (el) is placed in the scala tympani. The RD can hardly be seen on the superior surface of the OSL. RM, Reissner's membrane; ST, scala tympani; RW, Round window.

To minimize damage during CI, it is important that the electrode is retained within the ST and that the integrity of the endolymph space is maintained. The surgical area at RW insertion is located ~2.7 mm from the rim of the saccule membrane. At AICO and ACO, this distance is longer, but the risk for breaking the endolymph barrier is higher. Synchrotron imaging shows that the saccule wall consists of a thin and a thick portion. The thick portion lies near the bony margins of the spherical recess, and the thin portion faces the middle ear. The latter shows extreme fragility and may protect saccular receptors from high-energy stapes vibrations (32). This portion may be damaged or ruptured even by forceful mechanical pressure changes such as the “cork effect” at stapes removal. Entering the vestibular scala during cochleostomy increases the risk of bone dust entering the vestibule, which may lead to acute pro-inflammatory reactions and contribute to symptom manifestations. Moreover, the vibration produced by the milling process may cause statoconia dislocation and consequent vertigo. It may even explain benign positional vertigo, transient dizziness (33), and EH caused by dislocated saccular statoconia in the RD (34) (Figure 10). Therefore, direct drilling on the cochlear capsule should probably be kept to a minimum.

**Figure 11:**
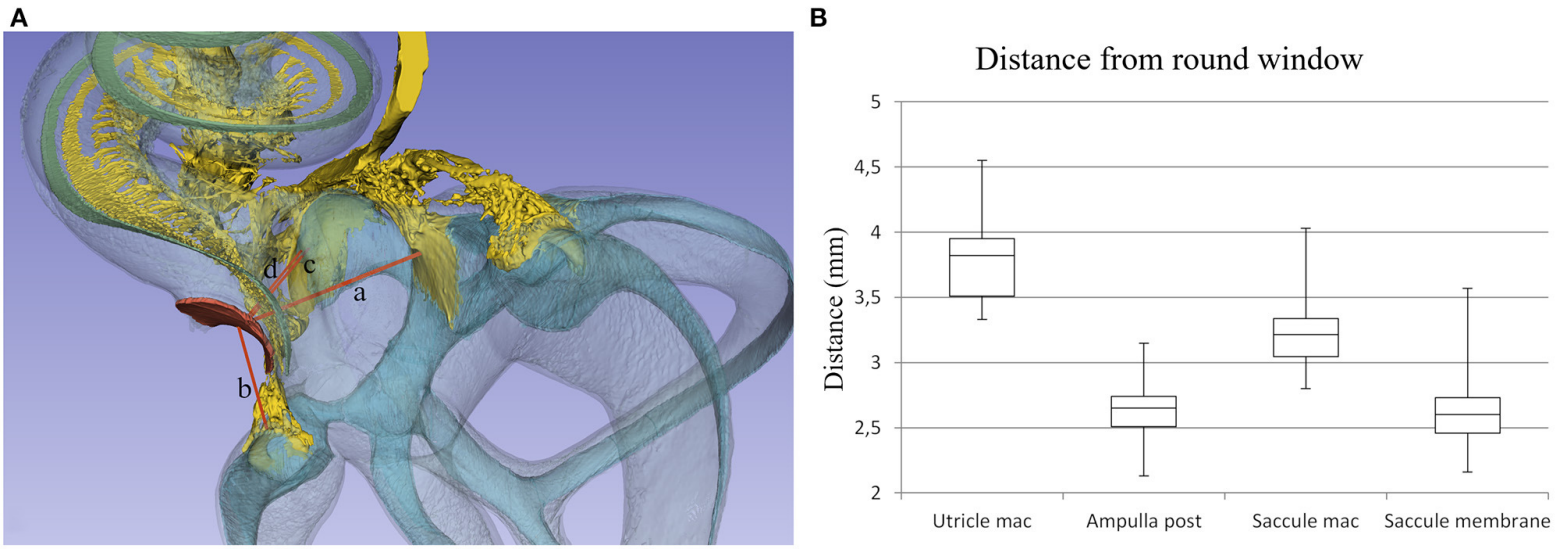
**(A)** Distances from the (a) utricle macula, (b) posterior semicircular canal ampulla, (c) saccule macula, and (d) saccule membrane to the middle of the RW were assessed. **(B)** Box plot showing measurements in 22 temporal bones.

There are other explanations for acute or persistent dizziness following CI surgery, such as fistulae in patients with large vestibular aqueduct syndrome (LVAS) (35) or EH (7, 36). The saccular receptors seem particularly vulnerable, reflected by changes in vestibular-evoked myogenic potentials (VEMPs) (37). Alterations such as new bone formation, vestibular fibrosis, saccule membrane distortion, and sub-epithelial thickening were described in studies where the CO technique was mostly performed (8). The authors suggested that the saccule is at greater risk for damage than the utricle or semicircular canals. According to Todt et al. (38), CO may degrade saccular function demonstrated by affected VEMP, and this was correlated with persistent dizziness. Similar results were noted by Jin et al. (39) studying 12 children undergoing CI and by Meli et al. in adults showing lack or reduction of VEMP responses (40). Licamelli et al. (41) found a majority of patients had vestibular impairment with altered saccular function indicated by VEMP as well as reduced vestibule-ocular reflex (VOR) gain. Our 3D study revealed the small distance between the most proximal region of the RW and the saccule (Figure 11), which may suggest that this region of the RW should be avoided during surgery. In some children with inner ear dysplasia, VEMP responses were also observed at electrical stimulation, suggesting that the vestibular nerve may be stimulated (39). This may be explained by the posterior ampulla and nerve positioned near the RW (Figure 11, Supplementary Tables 1, 2).

Optimal preservation of residual hearing requires a more atraumatic CI surgery which can be expected to diminish injury to the vestibular organ as well. However, there are indications of some damage to the vestibular receptors of the otolith organs and semicircular canals even when using soft surgery techniques (42). Insertion speed was found to influence hearing preservation and vestibular function. A slow electrode insertion speed seemed to facilitate complete insertion, and improved preservation of residual hearing and vestibular function after CI (43). Fortunately, patients with vertigo usually undergo central vestibular compensation and recover with little or no postural deficit (40). However, it has not been determined whether the surgical approach and design of electrodes influence the prevalence of vestibular problems. Synchrotron 3D analyses show that the RW approach may be less damaging to the inner ear compared with CO (15, 23), which is in accordance with the vestibular results obtained by Todt et al. (38). Batuecas-Caletrio et al. (44) found the RW approach safer and less traumatic than CO. However, no correlation between the surgical approach and occurrence of postoperative vertigo was found by Veroul et al. (45) or by Nassif et al. (46), who investigated children. Rah et al. (7) found that the RW approach resulted in less postoperative dizziness, but this was not statistically significant due to the small numbers of RW insertions. Hänsel et al. (47) performed a meta-analysis and showed a low incidence of postoperative vertigo, but it was slightly higher in the CO group compared with the RW group. A CO closer to the RW was said to reduce the BM penetrations (48). In our opinion, it is difficult to foresee the extent of the damage that may occur from using the CO technique even if drilling is performed far inferiorly near the acoustic crest at the RW (22, 49). It may appear possible to directly enter the ST, however due to the surgical angle and curved outline of the SL, it may not actually be the case. Nonetheless, there may be anatomical limitations that necessitate a CO, such as facial recess exposure, cochlear malformations, and angles reducing the visibility of the RW.

CI can also influence horizontal semicircular canal function, and the video head impulse test (vHIT) and caloric test have been recommended for a complete vestibular analysis (50). RW surgery may change canal and otolith organ function, as shown by Dagkiran et al. (51). They found that the posterior and superior semicircular canal functions were more affected than the lateral canal, recommending the use of a test battery capable of evaluating all five vestibular end-organs pre- and postoperatively. In a recent study in patients undergoing unilateral or bilateral CI, there was no significant impairment of lateral semicircular canal function as demonstrated by high-frequency VOR and vHIT compared with normal hearing individuals in the long term (46, 52). According to Nassif et al. (46), vHIT results suggest there is little impairment of LSSC function compared with normal hearing children (52). From an anatomical standpoint, a functional deterioration of the lateral and horizontal canals is likely to be caused by an indirect trauma caused by perilymph drain or contamination at surgery. Interestingly, SR-PCI revealed that the vestibular membrane apparatus is anchored by several gracile tissue pillars reaching the interior surface of the bony labyrinth. A massive drain of perilymph could rupture this fine network and lead to organ displacement and vestibular dysfunction. These findings may further point to the importance of a slow electrode insertion to minimize perilymph displacement and allow adaptation inside the scala and vestibule to reduce trauma.

Today, most congenitally deaf children receive implants in both ears. Vestibular concerns may arise if the patient is operated on in both ears simultaneously, or in the only vestibular functioning ear. Signs of damage to the saccule with loss of VEMP are common but seemingly with a limited correlation to vertigo, possibly due to transient disturbances (53) and central compensation (37, 41, 54). Colin et al. (4) prospectively tested vestibular function, using pre- and postoperative neuro-vestibular examination and clinical tests, and found no correlation between postoperative test results and postoperative vertigo. Occasionally, there was even improved balance following electric stimulation (4, 55, 56).

The present results using SR-PCI and micro-CT imaging three-dimensionally display the intriguing and difficult anatomy of the base of the cochlea and vestibular end-organs. This study may hopefully contribute to a better understanding of the spatial organization, thereby increasing surgical safety. Enhancement of surgical techniques, approaches, and design of CI electrodes may further lessen surgical trauma in the future.

## Data Availability Statement

The data supporting the conclusions of this article will be made available by the authors, upon request to the corresponding author.

## Ethics Statement

The study was approved by Western University, London, Ontario, Canada, in accordance with the Anatomy Act of Ontario and Western's Committee for Cadaveric Use in Research (approval no. 06092020).

## Author Contributions

GR and JS performed micro-CT of human cadavers. HML and JS performed image processing and 3D visualization of scanned objects provided by SA, HL, SR, and JS. HR-A and NS-M planned the project. Microdissections with cochleostomies were provided by FA and HR-A. HR-A, SA, and HL analyzed the images and wrote the manuscript.

## Conflict of Interest

MED-EL Medical Electronics, R&D, GmbH, and Innsbruck, Austria provided salary support for one research group member (HL) in accordance with the contract agreement with Uppsala University, Sweden 2018. The remaining authors declare that the research was conducted in the absence of any commercial or financial relationships that could be construed as a potential conflict of interest.

## Abbreviations

ACO: Anterior cochleostomy
AICO: Anterior-inferior cochleostomy
BM: Basilar membrane
CA: Cochlear aqueduct
CI: Cochlear implantation
CO: Cochleostomy
Dice-CT: Diffusible iodine-based contrast-enhanced computed tomography
EH: Endolymphatic hydrops
IAC: Internal acoustic canal
ICO: Inferior cochleostomy
ICV: Inferior cochlear vein
I_2_KI: Lugol's iodine solution
LSSC: Lateral semicircular canal
LVAS: Large vestibular aqueduct syndrome
Micro-CT: Micro-computed tomography
OSL: Osseous spiral lamina
OW: Oval window
PSSC: Posterior semicircular canal
RD: Reunion duct
RM: Reissner's membrane
RW: Round window
SG: Spiral ganglion
SL: Spiral ligament
SR-PCI: Synchrotron radiation phase-contrast imaging
ST: Scala tympani
VEMPS: Vestibular-evoked myogenic potentials
vHIT: Video head impulse test
VOR: Vestibule-ocular reflex.
