# Supplementary material for: Vestibular Organ and Cochlear Implantation–A Synchrotron and Micro-CT Study
Source: Front Neurol. 2021 Apr 7;12:663722. doi: 10.3389/fneur.2021.663722 (PMC8058461; doi:10.3389/fneur.2021.663722)
Supplement: Supplementary file 1 [file Data_Sheet_1.docx]

**SUPPLEMENTARY MATERIAL**

| Bone ID | Utricle macula | Ampulla post | Saccule macula | Saccule membrane |
| --- | --- | --- | --- | --- |
| 1R | 3.38 | 2.7 | 2.99 | N/A |
| 2L | N/A | 2.74 | 3.09 | N/A |
| 2R | 3.83 | 2.51 | 3.36 | N/A |
| 4L | 3.83 | 2.83 | 3.56 | N/A |
| 9L | 3.92 | 2.91 | 3.23 | N/A |
| 10L | 3.72 | 3.04 | 3.47 | N/A |
| 1512L | 3.65 | 2.51 | 2.83 | 2.6 |
| 1512R | 3.33 | 2.38 | 2.89 | 2.34 |
| 1526R | 4.15 | 2.43 | 3.67 | N/A |
| 1537L | 4.13 | 2.71 | 3.2 | 2.46 |
| 1540R | 4.07 | 2.23 | 3.3 | 2.73 |
| 1552R | 3.57 | 2.72 | 3.23 | 2.57 |
| 1563L | 3.8 | 2.13 | 3.23 | 2.96 |
| 1563R | 3.95 | 2.4 | 3.18 | 2.16 |
| 1564L | 3.51 | N/A | 3.09 | 2.54 |
| 1571L | 3.41 | 2.65 | 3.24 | 2.96 |
| 1621R | 4.23 | 2.62 | 3.35 | 2.61 |
| 1629R | 3.82 | 2.6 | 3.15 | 2.64 |
| 1637L | 3.37 | 2.6 | 2.8 | 2.44 |
| ME02 | 3.5 | 2.65 | 2.8 | N/A |
| ME04 | 3.83 | 2.78 | 3.03 | N/A |
| ME05 | 4.55 | 3.15 | 4.03 | 3.57 |
| Average | **3.79** | **2.63** | **3.21** | **2.66** |
| SD | **0.32** | **0.25** | **0.29** | **0.35** |

**Supplementary Table 1.** Distances from the utricle macula, posterior semicircular canal ampulla (Ampulla post), saccule macula, and saccule membrane to the middle of the RW were measured in mm (Observer 1). The saccule membranes were collapsed in many bones and thus could not be visualized or measured. N/A: Not available.

| Bone ID | Utricle macula | Ampulla post | Saccule macula | Saccule membrane |
| --- | --- | --- | --- | --- |
| 1R | 3.59 | 2.81 | 3.24 | N/A |
| 2L | N/A | 2.81 | 3.61 | N/A |
| 2R | 3.89 | 2.93 | 3.44 | N/A |
| 4L | 3.77 | 2.66 | 3.46 | N/A |
| 9L | 3.61 | 2.44 | 2.75 | N/A |
| 10L | 3.94 | 2.64 | 3.57 | N/A |
| 1512L | 3.42 | 2.52 | 2.48 | 2.48 |
| 1512R | 3.49 | 2.46 | 2.84 | 2.76 |
| 1526R | 4.24 | 2.62 | 3.67 | N/A |
| 1537L | 4.46 | 2.6 | 3.1 | 2.76 |
| 1540R | 3.94 | 2.21 | 3.17 | 3.12 |
| 1552R | 3.36 | 2.76 | 3.1 | 2.85 |
| 1563L | 3.67 | 2.07 | 3.39 | 2.85 |
| 1563R | 3.82 | 2.39 | 3.05 | 3.05 |
| 1564L | 3.22 | N/A | 3.2 | 2.51 |
| 1571L | 3.69 | 2.55 | 3.32 | 2.99 |
| 1621R | 4.32 | 2.73 | 3.56 | 2.98 |
| 1629R | 3.87 | 2.52 | 2.84 | 2.71 |
| 1637L | 3.66 | 2.67 | 2.98 | 2.52 |
| ME02 | 3.37 | 2.74 | 2.76 | N/A |
| ME04 | 3.67 | 2.9 | 3.41 | N/A |
| ME05 | 4.33 | 3.03 | 4.27 | 3.89 |
| Average | **3.78** | **2.62** | **3.24** | **2.88** |
| SD | **0.34** | **0.23** | **0.39** | **0.37** |

**Supplementary Table 2.** Distances from the utricle macula, posterior semicircular canal ampulla (Ampulla post), saccule macula, and saccule membrane to the middle of the RW were measured in mm (Observer 2). The saccule membranes were collapsed in many bones and thus could not be visualized or measured. N/A: Not available.

**Supplementary Video 1.** Electrode path simulation of a round window implantation of a CI-electrode in a micro-CT based 3D reconstructed human temporal bone.
